# A randomised trial of a 5 week, manual based, self-management programme for hypertension delivered in a cardiac patient club in Shanghai

**DOI:** 10.1186/1471-2261-8-10

**Published:** 2008-05-06

**Authors:** Feng Xue, Wen Yao, Robert J Lewin

**Affiliations:** 1 Postgraduate Area, 2nd Floor, HYMS, University of York, York, UK; 2 Centre for Disease Control, Hong Kou District, 197 Chang Yang Road, Shanghai, PR China; 3 British Heart Foundation Care and Education Research Group, Department of Health Sciences, Seebohm Rowntree Building, University of York, University Road, York, UK

## Abstract

**Background:**

In Shanghai there are 1.2 million people with hypertension, many of whom have difficulty in affording medical treatment. Community based, anti-hypertensive clubs have been created to provide health education but education alone is often ineffective. Lifestyle change programmes have shown some potential for reducing blood pressure but in previous trials have required specialist staff and extensive contact. We have previously demonstrated that self-management programmes delivered by health professionals, such as a nurse who has had short training in self-management techniques can change health behaviour and reduce symptoms. This study was designed to evaluate the benefits of a simple, cognitive-behavioural, self-management programme for hypertension based around a hypertension manual and delivered in the setting of a community anti-hypertensive club in Shanghai.

**Method:**

The method was a pragmatic randomised controlled trial with an intention-to-treat analysis. Adult patients with mild-to-moderate primary hypertension, waiting to join a neighbourhood anti-hypertension club, were randomised to the self-management programme or to an information only control procedure. They attended the group treatment sessions on 4 occasions over 5 weeks for education combined with goal setting for lifestyle change and an introduction to exercise. The main outcome measures were: changes in blood pressure; blood total cholesterol; diet; activity level and health related quality of life 1 month and 4 months after the end of treatment.

**Results:**

A total of 140 adults with mild-to-moderate primary hypertension took part. All of the main outcomes showed beneficial changes. Four months after the end of treatment the mean blood pressure differences between groups were systolic 10.15 mm Hg (P < 0.001, 95% CI 7.25–13.05), and diastolic 8.29 mmHg (P < 0.001, 95% CI 6.71–9.88). Patients in the intervention group also had significantly reduced weight, lowered blood total cholesterol, increased physical activity and improved quality of life.

**Conclusion:**

Patients with mild-to-moderate primary hypertension attending a 5 week, group and manual based, cognitive-behavioural self-management programme, delivered through a voluntary club in Shanghai experienced a significant reduction in blood pressure.

**Trial registration:**

Current Controlled Trials ISRCTN73114566

## Background

Hypertension is associated with a number of health behaviours such as lack of exercise and an unhealthy diet. Trials of lifestyle change programmes have shown worthwhile improvements in blood pressure (BP). For example the PREMIER trial, after subtracting change in the control group, showed a net reduction of 3.7 mmHg (P < 0.001) in systolic BP at 6 months and 4.3 mmHg (P < 0.001) in a second group of patients having additional counselling about an anti-hypertensive diet (the DASH diet) [1]. At 18 months the differences were not statistically significant [2]. In the ADAPT trial in Australia, positive changes in lifestyle and blood lipids were observed; however, the study appeared not to have reported the effect of its intervention on BP [3]. Both trials involved intensive contact with patients, long-term follow up and multi-professional input. For example, in the PREMIER trial patients attended 18 group sessions over 6 months plus 4 individual training sessions. Some cognitive-behavioural studies have targeted stress as a potential risk marker but results from such programmes have been mixed. A study by Linden [4] demonstrated that individualised stress management was associated with ambulatory BP reductions; however some other studies, e.g. Johnston's [5], have failed to replicate this effect. According to Mancia [6], stress may be linked to hypertension through increasing unhealthy behaviours. Reviewing these studies the 2004 Canadian recommendations for the management of hypertension recommended its use only in selected individuals [7].

In Shanghai the prevalence of hypertension has been rising for almost two decades; in 1991 it was 13%, in 1998 17% and in 2001 19% [8]. In 2006, 29.8% of over-35s in Shanghai were hypertensive [9]. The majority of Chinese patients are unable, or unwilling, to pay for expensive 'western' medicines for hypertension. Instead many use local cheap herbal medicines [10,11], for example the *zhenju *pill, a compound of chrysanthemum, pearl and a diuretic. There are believed to be 1.2 million hypertensive people in Shanghai [8]. As they get older there is likely to be an increase in the number of strokes and heart disease which will have a profound effect on the health of the population. In response a community based chronic disease management strategy has been adopted. One aspect of this strategy has been the establishment of voluntary and very popular 'hypertension and cardiac rehabilitation clubs'. Patients who join the clubs have public lectures on hypertension and lifestyle change with 40 or 50 people attending each time.

Educational interventions are known to be a relatively weak method for behavioural change when compared with cognitive-behavioural self-management programmes [12]. Unfortunately there are many patients to treat and few health professionals trained in cognitive-behavioural methods. One solution for delivering cognitive-behavioural self-management programmes to large numbers of people is the use of self-help manuals 'facilitated' by brief contacts with a health professional or lay person who has had short training in the cognitive-behavioural methods employed in the programme. Examples include the Heart Manual for delivering post-MI (myocardial infarction) cardiac rehabilitation which has been evaluated in several trials versus both routine care [13] and multi-disciplinary hospital based programmes [14] and has been found to be superior to the former and equal to the latter. In the UK the National Institute for Clinical Excellence has recently recommended the Heart Manual as being equivalent to a multi-disciplinary phase III hospital based rehabilitation programme and it is being widely used for that purpose. A similar self-management programme, the Angina Plan reduced episodes of angina by 40% and improved a number of health behaviours when delivered through 4 brief contacts with a nurse [15] and this too is being increasingly adopted across the UK.

In the study reported here we developed and evaluated a 5 week, group and manual based, cognitive-behavioural self-management programme for hypertension that could be delivered in the setting of a community based 'hypertension and cardiac rehabilitation club' in Shanghai.

## Methods

### Study participants

The study was conducted in a community anti-hypertensive club in Tilanqiao Neighbourhood, Shanghai. A chronic disease management system had been established in this neighbourhood as part of the work of the Tilanqiao Community Health Services Centre (CHSC). In this system all patients with a chronic disease receive regular health checks, the frequency of these depending on the severity of the illness. The checks are administered by community 'doctors' who are health workers trained in public health but have no right to prescribe medicine. For hypertensive patients, community doctors go to their homes at regular intervals to measure blood pressure and give simple advice about managing the disease.

The research subjects were adults with mild-to-moderate primary hypertension with no evidence of serious co-morbidity such as diabetes and angina. According to the 2003 European Society of Hypertension guidelines for the management of arterial hypertension [16], such patients need to be monitored for at least 3 months with only non-pharmacological treatment rather than begin drug treatment promptly. Hypertensive patients with concomitant diabetes, even if they have only high normal BP, are classified as at high risk, and should initiate immediate anti-hypertensive drug treatment which would have confounded the results of the trial. We had intended to recruit patients not having started drug therapy; however this was not available in the system, as all patients had been on medication, although the majority were taking herbal medicine. Furthermore, under the chronic disease management system in force in this neighbourhood diabetic patients were part of a different monitoring mechanism. Therefore diabetic patients were not included in the trial.

The inclusion criteria were:

• clinician diagnosed mild-to-moderate primary hypertension defined as having systolic BP between 140 and 180 mm Hg and/or diastolic BP between 90 and 110 mmHg in accordance with patients' medical records;

• patients aged 18–69 years: this was because the physical activity questionnaire we used was only valid in this range.

The exclusion criteria were:

• secondary forms of hypertension (e.g. hypertension resulting from renal disease);

• target organ damage and/or diabetes;

• congestive heart failure;

• angina;

• other life-threatening co-morbidity (e.g. carcinoma, terminal liver or renal failure);

• disability that would prevent participation in a walking exercise regime;

• an inability to read or communicate in Chinese and/or a history of reduced cognitive ability.

### Recruitment and randomisation

The names of the patients who satisfied the entry criteria were taken in a serial fashion from the list of patients being actively followed up in the chronic disease management system. The principle investigator (FX), accompanying a community doctor, conducted home visits and explained the study to these patients. If interested in taking part, they were given an information sheet and contacted again by telephone after one week. If still willing to take part, they were then invited to the CHSC for a baseline assessment. Informed consent was obtained, following which they were randomised by an independent community health doctor who was not involved in the research in any other way. The research subjects automatically became members of the Tilanqiao Community Anti-Hypertensive Club.

The patients were randomised to either the self-management programme or an information only control group with the promise that they would receive the intervention once the study was completed. Randomisation codes were generated by STATA and the person who executed the allocation sequence was blinded to the patients and took no other part in the research.

### The intervention

*The self-management group *intervention was delivered in 4 small group sessions spread over 5 weeks, the final session taking place two weeks after the third session. Each small group comprised 10–12 patients, there being 6 groups in all. Each of the first three sessions lasted 2.5 hours, and the final session around 1 hour. Table 1 presents the content of each session.

**Table 1:** contents of the cognitive-behavioural self management programme for hypertension by session

Day 1 in Week 1 (9.00–11.30)	Day 2 in Week 2 (9.00–11.30)	Day 3 in Week 3 (9.00–11.30)	Day 4 in Week 5 (9.00–10.00)
• Basic knowledge of hypertension	• Feedback from practice of goals	• Feedback from previous goals	• Feedback from previous goals
• Introduction to goal setting	• Physical activity, DASH diet, salt, alcohol, smoking	• Managing the pills	• Patient led exercise session
• Group exercise – using digital BP meter	• Patient led exercise session	• Patient led exercise session	• Maintaining change
• Handing out booklets 2–5	• Group exercise – calculating BMI	• Group exercise – food energy calculation	• Setting new goals
• Setting initial goals	• Setting new goals	• Setting new goals	

The programme followed a common format, consisting of:

• An educational talk, in accordance with a self-management manual (the Hypertension Manual), from a community doctor who was trained by the principal investigator (FX) in the methods being used in the programme. The community doctor also led three group exercises, which were, respectively, using digital BP meter, calculating body mass index (BMI), and estimating food energy.

• Goal setting for behaviours that would reduce hypertension such as improved diet or a higher level of activity. From the second session onwards patients reported back to their group the progress they had made with their individually set goals and received praise and encouragement from the group members before setting further goals. Between each session every patient was contacted by a 'group facilitator' (one of the group members) who phoned the others to check progress with the 'homework' goals that the patient had set in the group session. This facilitator was chosen by the principle investigator (FX) using his subjective judgement as to which patient would be most suitable for this role.

• A patient led exercise session. In each group one patient volunteered to give demonstration and then lead an exercise session in a form of exercise they knew well; in most cases this was *tai chi *or fan dance.

Self-monitoring was known to improve health behaviour and patients were provided free of charge with a digital BP meter, a weight scale, and a measuring tape to record daily BP, as well as weekly weight and waist circumference.

Patients were given a copy of the Hypertension Manual, which consisted of 5 separate booklets handed out at the first session.

• Booklet 1 gave basic knowledge about the causes of hypertension and dealt with a number of common misconceptions about hypertension. It also explained goal setting techniques and the self-monitoring of BP using the equipment provided.

• Booklet 2 gave information about desirable lifestyle changes including smoking cessation, increased levels of activity, weight reduction, controlling alcohol consumption to moderate levels, reducing the use of dietary sodium, and details of the DASH eating plan that was previously shown to reduce BP.

• Booklet 3 concerned compliance with medication and the maintenance of behaviour change.

• Booklet 4 was a diary for recording their daily BP as well as weekly weight and waist circumference.

• Booklet 5 was the 'Action Plan', a set of goal sheets on which the patients set out and recorded their success with their weekly goals.

The text of these booklets is available in both English and Chinese from the principal investigator (FX).

The main hypothesised therapeutic components of the intervention were: increasing activity levels, a reduction in or cessation of smoking, reduced sodium consumption and improved diet.

The main therapeutic methods employed were: education about the illness and changing unhelpful misconceptions, goal setting, group facilitation, self-monitoring and self-recording.

*The control group *received a booklet that was given to all members of the anti-hypertension clubs explaining desirable behavioural change and giving important information about hypertension. At the completion of the study the control group also took part in the self-management programme.

*Both groups *also received usual care as part of the chronic disease management programme.

### Sample size

The following assumptions were made: that the standard deviation of diastolic BP was 10 mmHg [17] and a reduction of blood pressure by 10/5 mmHg would be a clinically significant improvement [18]. Previous research showed that the DASH eating plan could lead to a reduction in diastolic pressure of 5.5 mmHg [19] and a reduction of 5–10% of body weight could reduce systolic and diastolic blood pressure by between 4–7 and 3–6 mm Hg respectively [20]. It was therefore thought reasonable to predict a mean diastolic blood pressure reduction of 5 mm Hg might be achieved. Requiring 80% power to detect such a change at α = 0.05 level would require 126 patients (63 in each group). Allowing for an attrition rate of 10% of patients over the course of the study yielded a recruitment target of 70 patients for each arm of the trial.

### Outcome measures

Baseline data was collected during the initial screening visit. Follow up data was collected on 2 occasions after treatment, the 1 st follow-up being 1 month after the end of treatment and the 2nd follow up being 4 months after the end of treatment. Staff blind to randomization collected and scored all measurements.

*Blood pressure measurements *were obtained by trained, certified individuals who used a mercury sphygmomanometer. After the participant sat quietly for 5 minutes, the observer measured BP in the right arm with an appropriately sized cuff. At each visit, 2 BP measurements separated by at least 30 seconds were obtained. A reading of systolic BP was taken when the first Korotkoff sound appeared, and a reading of diastolic BP was taken when the Korotkoff sounds disappeared [21]. At each assessment point, BP was the mean of all available measurements.

*Weight, height and waist circumference *were measured using, respectively, a calibrated scale, a wall-mounted stadiometer and an inelastic tape maintained in a horizontal plane.

*Biochemical tests *were taken by a chief technician and analysed at a laboratory in a hospital that was not connected to the study in any other way. Urine sodium and potassium was measured using ion selective electrodes (ISE); and the cholesterol oxidase method was used to establish blood total cholesterol. The normal range for blood total cholesterol was 2.8–5.8 mmol/L; for urine sodium 130–260 mmol/24 h; for urine potassium 25–100 mmol/24 h. Urine sodium divided by potassium was equal to the urine sodium-to-potassium (Na/K) ratio.

#### Risk markers – physical activity, diet, smoking, drinking

Information about physical activity was collected using the international physical activity questionnaire (IPAQ) [22]. Data collected with the IPAQ was reported as a continuous measure. The volume of activity was computed by weighting each type of activity according to its energy requirements to yield a score in MET-minutes. Total Physical Activity MET-minutes/week was equal to the sum of Walking, Moderate and Vigorous MET-minutes/week scores. The IPAQ instruments had been validated in China [23]. Diet was assessed using a validated dietary questionnaire [24] asking about intake of food items in the last three months, based on which the daily intake of food groups (red meat, vegetables, fruits) was calculated. For smoking, we asked patients if they had smoked in the last 4 weeks. For drinking, we asked them if they had: (1) 1–4 drinks per week; (2) more than 5 drinks per week.

#### Health Related Quality of Life (HRQOL)

Quality of life was assessed using the Medical Outcomes Study Short Form 12 Health Survey (SF12), a widely used tool to measure quality of life. SF-12 was derived in the United States from the twelve questions of the SF-36. It provides scores in two domains: the Mental Component Summary (MCS) and Physical Component Summary (PCS) scales [25]. Possible scores range from 0 to 100 with higher scores indicating better quality of life.

### Statistical Analysis

An intention-to treat analysis was used with missing final scores being replaced with the last valid observation for that subject. Use of the last observation carried forward is a popular and simple practice in analysing clinical trial outcomes [26]. Variation in outcome between the six subgroups was tested by analysis of covariance (ANCOVA) within the intervention group only adjusting for baseline as well as for subgroup as a categorical factor. This was only significant in one (P < 0.05) out of the 26 analyses, suggesting that subgroup effects were not important, and it was therefore justifiable to use simple ANCOVA as a statistical method for removing the effect of the baseline variable in testing for intervention effects. Comparison of groups for changes in smoking and drinking were made using chi-squared tests. When making comparisons between the intervention and control groups, adjusted means were used. All statistical tests were two-tailed and had a significant level of 5% (α = 0.05).

The use of effective western medication was uncommon but many patients took herbal remedies, the effectiveness of which was unknown. To control for the possibility that any change in blood pressure outcomes observed were the result of changes in the extent of the use of medications, a categorical variable called 'drug use' was created to ascertain if there were systematic changes in the use of these medications at the 2^nd ^follow up assessment. Three outcomes were categorised; these were: 'patient reducing the dose', 'patient increasing the dose' and 'no change'. With blood pressure used as the dependent variable, baseline blood pressure as the covariate, and the variable 'drug use' as the fixed factor, we examined if a change in the use of medications between groups resulted in the change in blood pressure.

All statistical analyses were executed using SPSS 14.0 for Windows.

### Ethical approval

This trial was carried out as part of the doctoral research training programme at the Department of Health Sciences of the University of York, United Kingdom. All student research must be approved by the Research Governance Committee at the University of York. The Centre for Disease Control in Hong Kou district Shanghai also reviewed and approved the protocol.

## Results

### Baseline characteristics

**Figure 1:**
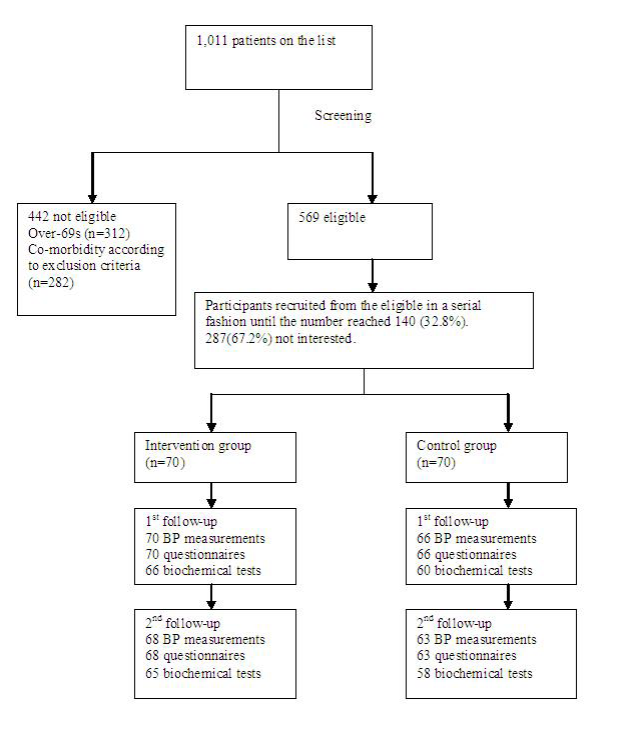
participant flow.

Recruitment began in November 2004 and ended in May 2005. The participant flow during the trial is shown in Figure 1:

Randomisation was successful and there were no significant differences between groups in any of the baseline measures (Table 2). In the intervention group, 53 (75.7%) people claimed they had obtained knowledge about hypertension in the past six months; and in the control group 58 (82.9%) people. There were only 24 smokers among the participants, 11 (15.7%) in the intervention group and 13 (18.6%) in the control group; and there were 28 drinkers, 17 (24.3%) in the intervention group and 11 (15.7%) in the control group. In the intervention group, 10 (14.3%) had more than 5 drinks per week; and in the control group, 5 (7.1%). All the participants had been taking medications and the majority (72.9%) were taking the *zhenju *pill. The numbers taking western drugs in the intervention and control groups were, respectively, 32 and 24; and the difference was not statistically significant.

**Table 2:** Baseline characteristics of participants by group

		Intervention group (n = 70)	Control group (n = 70)
*Demographics*			

Gender	Male (n)	29 (41.4%)	29 (41.4%)
	Female (n)	41 (58.5%)	41 (58.5%)
Age ^a^		57.5 (6.96)	57.4 (6.95)
Years after diagnosis^b^		7.50 (4.00,15.00)	8.50 (3.75,18.50)
Highest level of Education	Junior middle school and below	29 (41.4%)	24 (34.3%)
	Secondary technical school	8 (11.4%)	9 (12.9%)
	Senior middle school	12 (17.1%)	19 (27.1%)
	College (Associate)	14 (20.0%)	12 (17.1%)
	University (Bachelor)	7 (10.0%)	6 (8.6%)
Monthly income (yuan)	≥10000	1 (1.4%)	0 (0)
	5000–9000	3 (4.3%)	0 (0)
	2000–4000	9 (12.9%)	4 (5.7%)
	1000–2000	19 (27.1%)	31 (44.3%)
	≤1000	38 (54.3%)	35 (50.0%)

*Clinical indicators*			

BP (mm Hg)	Systolic ^a^	141.66 (12.81)	141.64 (12.65)
	Diastolic ^a^	89.70 (7.82)	89.43 (7.53)
Body mass index (BMI)^a^		25.17 (3.01)	24.74 (2.99)
Blood total cholesterol^a^(mmol/L)		5.74 (1.01)	6.00 (1.28)
Urine Na/K ratio^b^		2.11 (1.34–3.09)	2.40 (1.39–3.77)
Waist circumference^a^(cm)		85.87 (8.62)	83.61 (9.19)

*Quality of life*			

HRQOL SF-12	Physical domain ^a^	43.32 (7.59)	43.85 (7.53)
	Mental domain ^a^	51.87 (8.24)	53.49 (8.34)

*Behavioural indicators*			

Physical activity	Sitting total minutes/week ^a^	1676.29 (931.44)	1703.36 (895.74)
	Total physical activity ^a^		
	(MET-minutes/week)	3753.86 (2480.79)	3916.44 (2471.21)
Diet	Red meat (g) ^a^	60.01 (37.96)	52.99 (31.09)
	Vegetables (g) ^a^	320.77 (95.44)	320.05 (93.97)
	Fruits (g) ^a^	154.26 (86.44)	164.99 (91.72)

*Medications*			

*Zhengju *pill		43	59
Western medicine		32	24

^a^Mean (SD)

b Median (25–75th percentile)

### Intervention effects

The differences in outcomes for the two groups 1 month and 4 months after the end of treatment are summarised in Table 3.

#### Blood pressure

**Figure 2:**
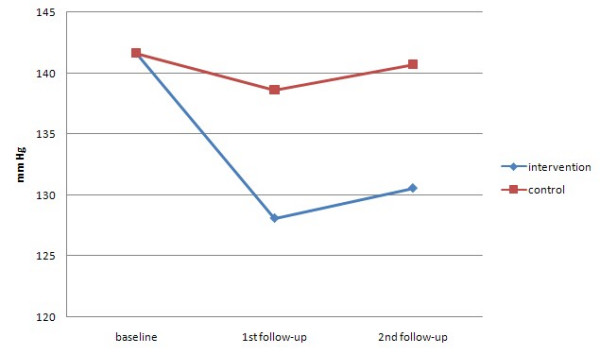
Mean systolic BP over time by randomised group.

Table 3 and Figure 2,3 present the differences between groups in blood pressure at both follow-up assessments. The intervention, but not the control group, experienced a significant reduction in both systolic and diastolic blood pressure at both assessments.

**Figure 3:**
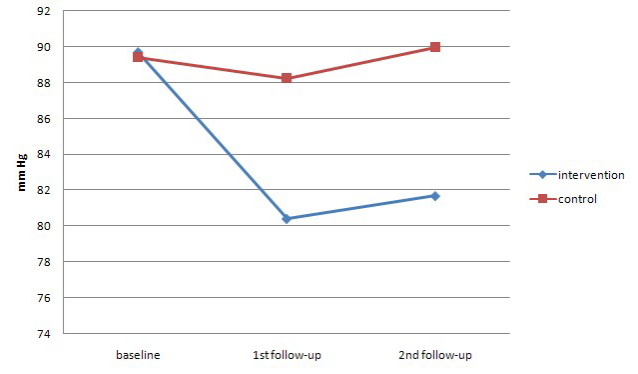
Mean diastolic BP over time by randomised group.

**Table 3:** Outcomes at 1st and 2nd follow-ups by group

Blood Pressure			Mean		Mean difference (95% CI)	P value
					
			Intervention	Control		
Systolic BP (mm Hg)		1st follow-up	128.10	138.62	10.52 (7.75–13.28)	<0.001
		2^nd ^follow-up	130.57	140.72	10.15 (7.25–13.05)	<0.001
Diastolic BP (mm Hg)		1^st ^follow-up	80.40	88.25	7.85 (5.98–9.72)	<0.001
		2^nd ^follow-up	81.67	89.96	8.29 (6.71–9.88)	<0.001
Total cholesterol (mmol/L)		1^st ^follow-up	4.99	5.43	0.45 (0.24–0.65)	<0.001
		2^nd ^follow-up	5.14	5.42	0.28 (0.06–0.49)	0.013
Urine Na/K ratio		1^st ^follow-up	0.67	0.60	0.07 (-0.26–0.11)	0.445
		2^nd ^follow-up	0.70	0.52	0.19 (-0.03–0.41)	0.094
Waist circumference (cm)		1^st ^follow-up	81.95	85.07	3.12 (2.36–3.89)	<0.001
		2^nd ^follow-up	82.80	85.17	2.37 (1.56–3.18)	<0.001
Body mass index (BMI)		1^st ^follow-up	24.39	24.72	0.33 (0.06–0.60)	0.016
		2^nd ^follow-up	24.43	24.74	0.31 (0.00–0.62)	0.048
Physical activity	1^st ^follow-up	Sitting total minutes/week	1238.35	1757.01	518.65 (374.46–662.85)	<0.001
		Total physical activity (MET-minutes/week)	4572.94	3475.49	1097.45 (900.81–1294.10)	<0.001
	2^nd ^follow-up	Sitting total minutes/week	1531.25	1844.42	313.18 (143.94–482.42)	<0.001
		Total physical activity (MET-minutes/week)	4259.58	3239.43	1020.15 (786.24–1254.06)	<0.001
Diet (daily intake)	1^st ^follow-up	Red meat (g)	59.10	57.63	1.47 (-2.47–5.41)	0.462
		Vegetables (g)	325.20	320.96	4.24 (-2.21–10.69)	0.196
		Fruits (g)	160.09	163.44	3.35 (-2.70–9.40)	0.276
	2^nd ^follow-up	Red meat (g)	60.45	58.72	1.74 (-3.59–7.07)	0.520
		Vegetables (g)	315.78	309.70	6.09 (-3.74–15.91)	0.223
		Fruits (g)	155.69	161.33	5.64 (-2.97–14.24)	0.197
HRQOL SF12	1^st ^follow-up	Physical	47.72	43.44	4.28 (1.85–6.71)	0.001
		Mental	56.48	52.66	3.83 (1.97–5.68)	<0.001
	2^nd ^follow-up	Physical	47.25	44.19	3.06 (0.72–5.40)	0.011
		Mental	54.88	52.80	2.08 (0.12–4.04)	0.038

#### Biochemical and clinical measures

Table 3 shows a similar pattern of findings for blood total cholesterol, BMI and waist circumference. The variable of urine Na/K ratio was not normally distributed. Logarithmic transformation was performed so that it could be analyzed using ANCOVA which showed that the differences were not significant.

#### Behavioural risk markers

The intervention group showed a much greater increase in physical activity than the control group but no significant differences existed between the two groups in dietary behaviours. For smoking and drinking, neither showed a significant difference in changes between the two groups. There were 9 (13.2%) smokers in the intervention group and 9 (14.3%) smokers in the control group 4 months after the end of treatment. In the intervention group 1 patient had quit smoking, and 1 smoker had dropped out of the study; in the control group 1 patient had quit smoking, and 3 smokers had dropped out of the study. In the intervention group 6 patients stopped having more than 5 drinks per week 4 months after the end of treatment, and in the control group 1 patient.

#### HRQOL

The mean differences in quality of life scores as measured by the physical and mental dimensions of the SF-12 were statistically significant with the intervention group having a clear advantage.

### Exploring interaction of drugs with BP

ANCOVA showed that changes in taking anti-hypertensive drugs could not explain the reduction of blood pressure (for SBP, F = 1.68, P = 0.19; for DBP, F = 1.38, P = 0.26). More people in the intervention group had reduced medication 4 months after the end of treatment, and the differences were statistically significant (P = 0.02) (Table 4). Also, there were no significant differences between the groups in the proportions taking western drugs at the end either in those taking at the start, 27 (84%) in intervention versus 19 (79%) in controls, or in those not taking at the start, 1 (3%) versus 3 (6%). Furthermore, the differences between the groups in final blood pressure (both SBP and DBP) remained at P < 0.001 even when one adjusted for the changes in medication in a multiple regression. Therefore the effect of the changes in use of medication over the trial on blood pressure can be excluded.

**Table 4:** Drug taking status at the 2^nd ^follow-up

Category	Control group	Intervention group
Patient s reducing the dose	10	23
Patients increasing the dose	6	2
Patient s taking same dose	54	45

χ2 = 7.94, P = 0.02

## Discussion

This study indicates that, at least in the short term, a simple cognitive-behavioural self-management intervention can lead to clinically significant reductions in both systolic and diastolic blood pressure. Patients also reduced their waist circumference, lost weight, became more active and reduced their blood cholesterol. Although previous trials of behavioural change programmes have produced similar benefits for hypertensive patients [1-3] this trial did so with a very much lower amount of input: only 8.5 hours of face to face contact and a simple self-help manual.

The most obvious and a serious limitation in this study is the short follow-up period. This was necessitated because of resource limitations. The benefits of cognitive-behavioural programmes are known to diminish over time [20] and further trials will be required before it can be assumed that this is a worthwhile treatment. In this study there was little evidence of regression in the blood pressure improvement between the first and second follow-ups but a more realistic follow-up period would be five years. If the medical benefits of taking part could be shown to be sustained over time, this simple programme might save many lives in China and possibly in other countries where lifelong treatment with 'western' medicines is not an option for the general population.

Another apparent limitation is that only around a third of those who were invited agreed to take part (Figure 1). Of course this proportion might increase if the method was known to be effective. From the demographic data it seemed it might have been people with better health behaviour who volunteered and self-evidently the people who took part were interested in trying to self-manage their blood pressure. Only 17% of the participants were smokers, 50% lower than the prevalence rate in Shanghai (36%) [27]. The fact that only a proportion of the population want to actively manage their health does not mean that this treatment should be ignored; if 30% of those in Shanghai with hypertension agreed to take part this would bring treatment to around 360,000 people.

One of the weaknesses of this and most other trials of cognitive-behavioural treatments is that patients are not blind to allocation and the control group may do worse because they know that they have been denied a treatment they were interested in. To try to minimise this problem, in this study the control group patients were told that they would receive the treatment when the research project was finished; and there was no evidence that their blood pressure went worse during the trial. It was also clear that their blood pressure remained the same and that all of the change observed was a reduction in the intervention group's blood pressure.

Small groups may vary in effectiveness because it is impossible to create the same circumstances for each group. In this study, for example, a 'group facilitator' was chosen by the principle investigator and that person's personality might have influenced the effectiveness of the group. However statistical tests showed that this was not the case and that results did not vary by group.

A potential complication in interpreting the results in this study was the degree of self-medication with popular herbal remedies or with western medicines. The effectiveness of the herbal treatment is controversial [10,11]. Even if we presumed the traditional medicine to be ineffective, there was not an imbalance between the groups for that factor. At baseline there was not a significant excess of people in the intervention group taking western medicine; and 4 months after the end of treatment there were also no significance differences between the groups in the proportions taking western drugs. The changes in BP may be even more surprising because they were also accompanied by a reduction in the use of medication, both Chinese and western, in the intervention group and a very slight increase in the controls.

The study did not achieve the hoped for benefits of changing dietary behaviour and the level of urine sodium. However, the participants appeared to already have had a reasonably good diet, and the urinary Na/K ratio was quite low (median = 2.14 at baseline) as compared with north China. Of the 52 centres in the Intersalt study, Tianjin, a city in north China, had the highest urinary Na/K ratio reaching as high as 6.7 [28]. Their dietary pattern was close to that recommended by the China Nutrition Society [29]. Weight, blood total cholesterol and waist circumference were all significantly reduced. The behaviour that did change significantly was activity levels. People in the intervention group were sedentary for less time and expended about 1,000 MET minutes more activity a week. Theoretically an increase in activity could be responsible for all of these benefits including the reduction in total cholesterol [30]. Applying certain psychological models, such as goal setting, to self-management programmes appears to be important elements for them to be successful [6]. Setting realistic and graduated goals was used in this study as a core behavioural change technique. Reaching goals would give patients a sense of control over their illness, which could lead to the setting of more rigorous goals at the next level.

Delivering treatments in countries where primary care services are not well developed is a challenge. In Shanghai the club-based model provides a good platform for chronic disease self-management, ensuring sustainability and accessibility. The results of this study suggest that this new approach could easily be integrated with the existing local chronic disease management system. Many community health services centres have already set up anti-hypertensive clubs; and it may be the case that they could have a more effective impact on hypertension if they adopted a cognitive-behavioural approach, whereby patients are engaged in practical exercises to change behaviour rather than only attending public lectures. Tilanqiao Neighbourhood has a population of 104,745, and 54 residents' committees [31]. It is unlikely that that the Tilanqiao club could provide help for all of the hypertensive patients in the local community and the Community Health Services Centre might consider setting up clubs at the residents' committee level to allow greater access. Health cadres at residents' committees could act as facilitators, and the Community Health Services Centre as a coordinating centre.

## Conclusion

In Shanghai, a simple intervention of 8.5 hours' face-to-face group contact and a self-help manual produced improvement in lifestyle accompanied by clinically significant reductions in blood pressure in patients with mild-to-moderate hypertension. Further research is required to see if the benefits would persist long enough to have an impact on the incidence of stroke and heart disease.

## Competing interests

The authors declare that they have no competing interests.

## Authors' contributions

FX with RL designed the intervention and the research protocol. FX directed the trial and analysed the data. WY selected the sampling frame and contributed to the design of the study. FX and RL wrote the initial draft of this paper. All authors read and approved the final manuscript.

## Pre-publication history

The pre-publication history for this paper can be accessed here:


